# The Dynamic Responses of Cell Walls in Resurrection Plants During Dehydration and Rehydration

**DOI:** 10.3389/fpls.2019.01698

**Published:** 2020-01-21

**Authors:** Peilei Chen, Niklas Udo Jung, Valentino Giarola, Dorothea Bartels

**Affiliations:** Faculty of Natural Sciences, Institute of Molecular Physiology and Biotechnology of Plants (IMBIO), University of Bonn, Bonn, Germany

**Keywords:** cell wall composition, cell wall signaling, transcriptomes, resurrection plants, dehydration, rehydration

## Abstract

Plant cell walls define the shape of the cells and provide mechanical support. They function as osmoregulators by controlling the transport of molecules between cells and provide transport pathways within the plant. These diverse functions require a well-defined and flexible organization of cell wall components, i.e., water, polysaccharides, proteins, and other diverse substances. Cell walls of desiccation tolerant resurrection plants withstand extreme mechanical stress during complete dehydration and rehydration. Adaptation to the changing water status of the plant plays a crucial role during this process. This review summarizes the compositional and structural variations, signal transduction and changes of gene expression which occur in cell walls of resurrection plants during dehydration and rehydration.

## Introduction

**Figure 1:**
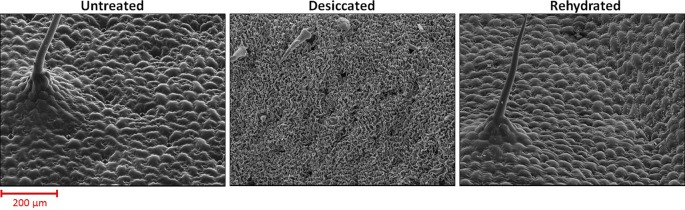
Surface images of Craterostigma plantagineum leaves taken with a scanning electron microscope. Micrographs were taken from untreated (RWC = 100%), desiccated (RWC = 2%) and rehydrated (RWC = 90%) leaves.

Plants as sessile organisms cope with environmental challenges by adopting a wide spectrum of strategies (Bartels and Salamini, 2001). Drought, a pervasive stress, causes water deficit (Bray, 1997) and may even lead to desiccation, a condition where only the bound water is left in the plant cells (Ramanjulu and Bartels, 2002; Zhang and Bartels, 2018). Although seeds of higher plants withstand desiccation (Bewley, 1979), vegetative tissues of most plants do not tolerate a water content which is below 60–30% (Challabathula and Bartels, 2013; Zhang and Bartels, 2018). However, some bryophytes, ferns, and a few angiosperms can survive in an extremely arid environment (Alpert, 2000). The desiccation tolerant plants, termed resurrection plants, can equilibrate their vegetative tissues with nearly 0% relative humidity (Gaff, 1971), stay in a dehydrated, quiescent stage for months, and resurrect once water is available again. Water loss leads to plasmolysis and subsequently causes mechanical stress (Moore et al., 2006; Plancot et al., 2019). Desiccation tolerant tissues can avoid or resist detrimental effects of this stress through increased vacuolation and/or cell wall folding (**Figure 1**) (Webb and Arnott, 1982; Moore et al., 2006; Farrant et al., 2007) which requires structural flexibility as well as physiological and molecular responses in the cell wall. In this review, we will focus on the changes in polysaccharide composition, cell wall signaling, and transcriptional changes, which are linked to reversible cell wall folding in resurrection plants (**Figure 1**).

## Dynamic Pectin Changes in Resurrection Plants Upon Desiccation/Rehydration

The cell walls encapsulate plant cells and provide mechanical strength. They define the morphology, and are implicated in plant growth and responses to environmental stresses (Pilling and Höfte, 2003; Hamann, 2014). Important building blocks of cell walls are cellulose, callose, pectin, and hemicelluloses. Pectin is the most abundant component and accounts for up to 50% (w/w) of the cell wall in *Arabidopsis thaliana* (Zablackis et al., 1995). Cellulose and callose are linear homopolysaccharides and are composed of β-(1,4)- and β-(1,3)-linked glucose residues, respectively. Cellulose microfibrils are interconnected by hemicelluloses and pectin and form rigid structures which build up the mechanical scaffold of the cell wall (Nishiyama, 2009; Wang et al., 2012). Pectin is a heterogenous matrix of homogalacturonan, rhamnogalacturonan-I, and rhamnogalacturonan-II. Homogalacturonan is typically most abundant and accounts for about 65% of pectin. Rhamnogalacturonan-I accounts for 20–35% and rhamnogalacturonan-II is a minor component (Mohnen, 2008). α-(1,4)-Linked D-galacturonic acid is a building block of homogalacturonan, where it is arranged in linear chains. Galacturonic acid is also the building block of rhamnogalacturonan-II and, together with rhamnose, the backbone of rhamnogalacturonan-I. Rhamnogalacturonan-I and rhamnogalacturonan-II are more complex than homogalacturonan, because galacturonic acid and rhamnose are substituted by other sugar residues. The biosynthesis of pectin has been reviewed recently (Harholt et al., 2010; Lampugnani et al., 2018) and will not be described further. Xyloglucan and xylan are the most abundant hemicelluloses in dicot cell walls and crosslink cellulose fibrils (Park and Cosgrove, 2015; Simmons et al., 2016). Xyloglucan has a β-(1,4)-linked glucose backbone with side chains which contain xylose, galactose (possibly acetylated), fucose, and arabinose. Xylan is made of β-(1,4)-linked xylose residues with side chains of α-arabinofuranose and α-galacturonic acid. Modifications such as transglucosylation, acetylation, or methylesterification and cross-linking of the different cell wall components play a major role in modifying the mechanical properties of plant cell walls (O’Neill et al., 2001; Ryden et al., 2003; Caffall and Mohnen, 2009; Caffall et al., 2009; Park and Cosgrove, 2015). Analyzing the behavior of the polysaccharide matrix in response to stress is essential to understand the flexibility of cell walls. Upon desiccation, the vacuole shrinks, and the cell contents are drawn inwards, which results in more tension between the plasmalemma and the cell wall (Levitt and Levitt, 1987). Callose synthesis is induced in response to different stresses and it functions as a local cell wall stabilizer (Nielsen et al., 2012; De Storme and Geelen, 2014). Upon desiccation most resurrection plants undergo extensive folding of the cell wall, a process which is quickly reversed during rehydration (Phillips et al., 2008; Jung et al., 2019). Controlled cell wall folding prevents tearing of the plasmalemma from the cell wall, which is essential to maintain cell integrity (Thomson and Platt, 1997; Farrant and Sherwin, 1998; Vicré et al., 1999; Farrant, 2000; Vicré et al., 2004b). The degree of folding depends on the leaf morphology and the leaf area e.g. the leaves of the desiccation tolerant grass *Oropetium thomaeum* (VanBuren et al., 2017) are narrow and the degree of folding is less than in *Craterostigma plantagineum*. Cells of desiccated leaves show the most extensive folding after dehydration compared to cells of roots or stems.

**Figure 2:**
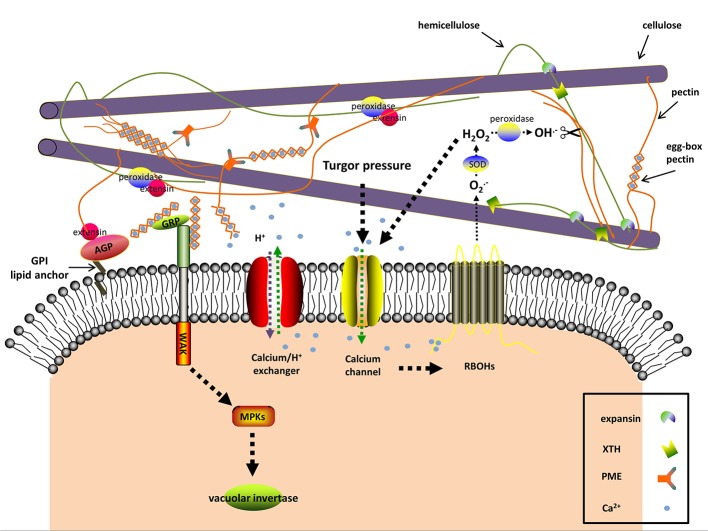
The predicted interactions among apoplastic proteins and signaling molecules in resurrection plants during dehydration. Dehydration induces turgor pressure changes, which are sensed by mechanosensitive (MS) calcium channels As a consequence [Ca^2+^]_cyt_ levels rise. The plasma membrane-localized NADPH oxidases (respiratory burst oxidase homologs, RBOHs) are activated through binding [Ca^2+^]_cyt_ and produce O_2_^−^, a substrate of the cell wall superoxide dismutase (SOD). The apoplastic H_2_O_2_ as the product of SOD also leads to Ca^2+^ influx. Cell wall peroxidases produce hydroxyl radicals (OH^−^) with apoplastic H_2_O_2_ as substrate. The reactive OH^−^ is able to rupture glycosidic bonds and leads to cell wall loosening. In addition to OH^−^, expansin and xyloglucan endotransglucosylase/hydrolase (XTH) may contribute to loosening the cell wall by disrupting the interaction between hemicellulose and cellulose during the early stages of dehydration and rehydration. Cell wall peroxidases facilitate wall stiffness by reinforcing the cross-linking of extensin with cell wall polysaccharides. Excessive [Ca^2+^]_cyt_ is toxic and thus [Ca^2+^]_cyt_ is transported to the extracellular space by Ca^2+^ efflux systems (Ca^2+^ exchangers are shown). The alkalization in the apoplast affects the activity of many cell wall proteins, one of which is pectinmethylesterase (PME). PME exerts its demethylesterifying role on pectin in alkalized apoplast and generates negatively charged pectins, which form the egg-box pectin gelatin with apoplastic Ca^2+^ and increase wall stiffness. Wall-associated kinase (WAK) forms a complex with the glycine-rich protein (GRP) and can detect the egg-box pectin. This then in turn activates the vacuolar invertase activity. The classic (glycosylphosphatidyl inositol) GPI-anchored cell wall arabinogalactan proteins (AGPs) are involved in signal transduction between the intracellular and extracellular compartments and act as plasticizers in resurrection plants against desiccation. The signaling pathway and the interactions in the apoplast are hypothesized according to the available literatures and the current research on cell walls of resurrection plants.

In resurrection plants, changes in homogalacturonan, rhamnogalacturonan-I, rhamnogalacturonan-II, and hemicelluloses were investigated in leaves of *C. plantagineum, C. wilmsii,* and *Lindernia brevidens* during dehydration and rehydration to understand cell wall plasticity (Vicré et al., 1999; Jung et al., 2019). Higher levels of de-methylesterified homogalacturonan were found upon desiccation which was reversed after rehydration. Homogalacturonan is synthesized in the methylesterified form and subsequently de-methylesterified in the cell wall, which suggests *de novo* synthesis of homogalacturonan during rehydration (Zhang and Staehelin, 1992; Staehelin and Moore, 1995; Sterling et al., 2001). A high proportion of de-methylesterified homogalacturonan upon desiccation in combination with calcium (Vicré et al., 1999) leads to the formation of the so-called “egg-box” structures (**Figure 2**) (Grant et al., 1973; Jarvis, 1984; Moore et al., 1986; Lloyd, 1991) which are proposed to strengthen the cell wall (Vicré et al., 1999; Jung et al., 2019). Highly de-methylesterified homogalacturonan provides additional binding sites for pectin binding proteins which might be important to sense the cell wall hydration status (Giarola et al., 2016; Jung et al., 2019). A role of homogalacturonan in desiccation tolerance is supported by a report that correlates accumulation of homogalacturonan with desiccation resistance in the green algae *Zygnema* sp. (Herburger et al., 2019). Changes in rhamnogalacturonan-I, rhamnogalacturonan-II, and the hemicelluloses may reinforce the cell wall upon desiccation in resurrection plants. In *C. wilmsii* and *C. plantagineum* the xyloglucan levels increased upon desiccation (Vicré et al., 1999; Jung et al., 2019). More xyloglucan points to an increase of interconnected cellulose fibrils and thus enhances cell wall rigidity (Moore et al., 1986; Fry, 1989; Park and Cosgrove, 2015). Xylan, another cellulose-linking cell wall component, is also increased upon desiccation but motile and flexible cell wall components like β-1,4-galactan and α-1,5-arabinan do not change (Jung et al., 2019). In *C. plantagineum*, dehydration leads to changes in rhamnogalacturonan-II (Jung et al., 2019). In the studied resurrection plants, the changes in the pectin composition lead to a more rigid cell wall upon dehydration (Vicré et al., 1999; Jung et al., 2019). Crosslinking of homogalacturonan *via* Ca^2+^ and rhamnogalacturonan-II *via* borate strengthens the cell wall (Kobayashi et al., 1996).

## Cell Wall Signaling in Resurrection Plants During Dehydration

The plant cell wall has a complex signaling system which monitors cell wall integrity by detecting chemical and physical modifications of the cell wall polymers and then transduces this information into the cell to trigger appropriate responses (Seifert and Blaukopf, 2010; Voxeur and Höfte, 2016). Signaling pathways resemble yeast signaling mechanisms and can be activated by various stimuli including drought stress (Hamann, 2014). Different molecules, including reactive oxygen species (ROS) and hormones, are integrated in cell wall-mediated signaling cascades (Miller et al., 2010; Choudhury et al., 2017; Novaković et al., 2018). An overview of the different physical and chemical signals and pathways is provided in the following paragraphs.

### Turgor Pressure and Turgor Sensors

Turgor pressure is the result of the osmotic pressure in the symplast and the mechanical strength of the cell wall (Cleland, 1971; Lewicki, 1998; Ringli, 2010). Altered turgor pressure can be perceived as physical signal by cell wall sensors such as ion channels, leading to the flow of Ca^2+^ between intra- and extracellular spaces according to the plasma membrane tension (Seifert and Blaukopf, 2010; Hamann, 2014; Hamilton et al., 2015) (**Figure 2**). Dehydration lowers the turgor pressure, thus stopping cell expansion and growth (Tardieu et al., 2014). The resurrection plant *Myrothamnus flabellifolia* maintains cell turgor and copes with the mechanical stress by increasing cell wall elasticity with plasticizers, i.e., arabinose-containing polymers (Moore et al., 2013). These polymers might act as “mechanosensors” as well (Moore et al., 2006; Moore et al., 2008; Le Gall et al., 2015).

Turgor pressure changes, as a result of dehydration, are perceived as mechanical signals by a specialized plasma membrane localized-mechanosensory gauge, termed mechano-sensitive or stretch-activated ion channel (**Figure 2**) (Seifert and Blaukopf, 2010; Hamilton et al., 2015). The pore-forming mechano-sensitive ion channels control ion passage *via* sensing the membrane tension and thus trigger the downstream signaling. In this way the change of mechanical force is sensed at the membrane-wall interface and acts as cell wall integrity sensor (Seifert and Blaukopf, 2010; Wolf et al., 2012; Basu and Haswell, 2017). In plants, three mechanosensitive channel families have been characterized, namely MscS-like channels, two-pore domain K^+^ channels, and Mid1-complementing activity channels (Hamilton et al., 2015; Basu and Haswell, 2017). The non-selective mechanosensitive-like channels are classified into three groups, among which the plastid-localized group II MscS-like 2 and MscS-like 3 are correlated with plastid osmotic stress and abscisic acid (ABA) induction. Overexpression of plasma membrane-localized group III MscS-like 10 can result in H_2_O_2_-associated cell death (Veley et al., 2014; Hamilton et al., 2015). The Mid1-complementing activity channels (Ca^2+^-permeable mechanosensitive channels) are tightly associated with Ca^2+^ influx and involved in regulating Ca^2+^ homoeostasis (Hamilton et al., 2015). The transcriptome analysis of the angiosperm resurrection plant *C. plantagineum* and the desiccation-tolerant lichen *Cladonia rangiferina* revealed elevated expression of genes encoding pore calcium channels and other non-defined ion channels upon dehydration (Rodriguez et al., 2010; Junttila et al., 2013; Giarola et al., 2015).

### Pectin-Derived Oligosaccharides

Many studies identified breakdown products of pectin as signaling molecules (Bidhendi and Geitmann, 2016; Cosgrove, 2016; Voxeur and Höfte, 2016). Pectin-derived oligosaccharides (OGAs) were first discovered in plant pathogen studies and belong to a class of elicitors, leading to damage-associated molecular patterns (DAMPs) or pathogen-associated molecular patterns (PAMPs), which are related to wounding or diseases (De Lorenzo et al., 2018; Nürnberger and Kemmerling, 2018). The cell wall surveillance system is able to distinguish the degree of polymerization and conformation of OGAs and to trigger different responses, accordingly (Cabrera et al., 2008; Osorio et al., 2008; Cabrera et al., 2010). In *A. thaliana* de-methylesterified pectin stretches bind to calcium and form so-called “egg-box” structures which are recognized by cell wall-associated protein kinases (WAKs) (Decreux and Messiaen, 2005). Dehydration leads to a higher level of de-methylesterified pectin and an increase in the concentration of calcium in the cell wall of the resurrection species *Craterostigma* (Vicré et al., 1999; Vicré et al., 2004a; Jung et al., 2019), which is the basis for the “egg-box” formation and the pectin-WAK association. These results support a role for OGAs as signal molecules in resurrection plants during water deficit (**Figure 2**). The xyloglucan-derived OGAs also regulate cell wall expansion (Pilling and Höfte, 2003; Seifert and Blaukopf, 2010). Fry et al. (1990) observed the modulating effects of xyloglucan-derived OGAs in plant growth. Takeda et al. (2002) proposed the involvement of the xyloglucan metabolism in cell elongation. During dehydration the structure and distribution of xyloglucan are significantly altered in resurrection plants (Vicré et al., 1999; Vicré et al., 2004a; Vicré et al., 2004b). Therefore, it is tempting to speculate that xyloglucan is involved in defense responses under dehydration, when other intrinsic defense systems are shut-down.

### Calcium

Calcium participates in multiple biological processes and has different functions in the cell wall. Besides a structural role in forming “egg-box” structures, calcium can move in and out of the cell and functions as second messenger (Parre and Geitmann, 2005; Bose et al., 2011; Kurusu et al., 2013). The majority of Ca^2+^ is localized in the apoplast and vacuole (Medvedev, 2005). In the apoplast, the excess of free Ca^2+^ is sequestered *via* the formation of “egg-box” structures (Voxeur and Höfte, 2016), which also serves as reservoir for cytosolic calcium ([Ca^2+^]_cyt_). Transient [Ca^2+^]_cyt_ elevation is a ubiquitous signal when plant cells encounter abiotic or biotic stress (Bose et al., 2011), which can be induced by OGAs (Moscatiello et al., 2006), and ascorbate (Makavitskaya et al., 2018). [Ca^2+^]_cyt_ elevation activates ROS production, and *vice versa*. ROS can also cause Ca^2+^ influx thus facilitating signal propagation (Seifert and Blaukopf, 2010; Kurusu et al., 2013) (**Figure 2**). Because Ca^2+^ reacts with proteins and other substances in the cytoplasm, high concentrations of [Ca^2+^]_cyt_ are detrimental. Therefore it is necessary to maintain the basal [Ca^2+^]_cyt_ levels with the help of cytosolic buffering systems and Ca^2+^ efflux systems (Ca^2+^-ATPases and Ca^2+^ exchangers) (Bose et al., 2011) (**Figure 2**). Repetitive Ca^2+^ influx and efflux give rise to cytosolic calcium oscillations, which vary in magnitude, frequency, and shape and are related to the severity and type of stress (Bose et al., 2011). Long term drought in soybean induced large Ca^2+^ efflux from mesophyll cells, accompanied by large K^+^ efflux and H^+^ influx, which may prime the ABA signal transduction in guard cells and finally lead to stomata closure (Mak et al., 2014). Similar to soybean, the apoplastic Ca^2+^ was also increased in the resurrection plant *C. wilmsii* upon dehydration, but with no significant change of K^+^ in the cell wall (Vicré et al., 2004a), which suggests that the resurrection plants may have a specific Ca^2+^ signaling mechanism. Mihailova et al. (2018) speculated that the accumulation of Ca^2+^ in the cell wall of *C. wilmsii* resulted from electrolyte leakage. This explanation may overlook the fact that neither the apoplastic K^+^ nor the phosphate significantly increased. The apoplastic Ca^2+^ in *C. wilmsii* was quantified using secondary ion mass spectrometry technology. However, the studies of Ca^2+^ signature require more real-time data and Ca^2+^ levels should be determined using microelectrode ion flux measurement and dynamic calcium imaging (Krebs et al., 2012).

### Protons

The proton influx and efflux across the plasma membrane can lead to apoplastic alkalization or acidification, which dictates the activities of pH-dependent cell wall modifying enzymes and finally affects cell wall structures. Water deficit, similar to other stresses such as salinity or pathogen infection tends to decrease proton concentrations in the apoplast (Geilfus, 2017). Increased apoplastic pH inhibits expansin activity and activates pectin-methylesterases, which together with elevated [Ca^2+^]_apo_ eventually strengthen the cell wall (Wolf et al., 2012) (**Figure 2**). The cell wall pH also varies spatially with a lower pH in the growing tip, thereby promoting cell wall loosening in apical tips (Moore et al., 2008; Mangano et al., 2018). Systemic apoplastic alkalinization is considered as a stress signal stimulating ABA accumulation in guard cells and stomatal closure during dehydration (Geilfus, 2017; Karuppanapandian et al., 2017). Therefore it is essential to consider the effect of pH on the activity of cell wall modifying enzymes in more detail.

### ROS and ROS-Producing Enzymes

Reactive oxygen species (ROS), including hydrogen peroxide (H_2_O_2_), hydroxyl radical (OH^−^), superoxide anion (O_2_^−^), and nitric oxide (NO) are a group of reactive molecules with partially reduced or active forms of oxygen (Choi et al., 2017). Historically ROS were only considered to be toxic for cell metabolism, but now it is widely accepted that ROS also act as important transmitters for both intra- and intercellular signaling (Hamann et al., 2009; Choudhury et al., 2017; Mittler, 2017). The apoplastic ROS trigger multiple downstream responses which demands a precise signal perception and transduction from the apoplast to the nucleus (Wrzaczek et al., 2013; Kangasjärvi and Kangasjärvi, 2014). The ROS signaling in the extracellular compartment has not been well deciphered. The predicted ROS sensing and transduction involve plasma membrane receptor-like kinases (RLKs), ion channels, aquaporins, redox balancing substances, plasma membrane lipid oxidation, and modification of cysteine residues in relevant proteins (Dynowski et al., 2008; Spoel and Loake, 2011; Kangasjärvi and Kangasjärvi, 2014). In resurrection plants not many studies on apoplastic ROS signaling exist. Based on observations of *Ramonda nathaliae* Jovanović et al. (2011) proposed that controlled production of ROS is a vital part in sensing dehydration and inducing multiple responses.

In plants, a considerable amount of ROS is generated intracellularly due to photosynthesis, mitochondrial respiration, photorespiration, and other processes caused by diverse stresses (Kimura et al., 2017; Mittler, 2017). In the extracellular space, the plasma membrane-localized NADPH oxidases (respiratory burst oxidase homologs) and cell wall peroxidases are the main sources for ROS production (Suzuki et al., 2011; O’Brien et al., 2012; Choudhury et al., 2017; Kimura et al., 2017). The respiratory burst oxidase homologs are activated *via* the influx of apoplastic Ca^2+^, internal Ca^2+^ binding, and phosphorylation (Baxter et al., 2014) (**Figure 2**). Under stress respiratory burst oxidase homologs are not only ROS producers, but also transmit ROS waves from one cell to neighboring cells (Choi et al., 2017). The apoplastic H_2_O_2_ is derived from spontaneous chemical reactions or superoxide dismutase-mediated mutation of superoxide which is generated from respiratory burst oxidase homologs (**Figure 2**) (Baxter et al., 2014), xanthine dehydrogenase (Ma et al., 2016), and oxalate oxidase (Voothuluru and Sharp, 2013). The comparative genome analysis of the desiccation tolerant lycophyte *Selaginella tamariscina* and the desiccation-sensitive *Selaginella moellendorffii* showed that the number of ROS-producing genes such as respiratory burst oxidase homologues and oxalate oxidase genes are much lower in the genome of the desiccation tolerant *S. tamariscina* compared to *S. moellendorffii* (Xu et al., 2018). This indicates that *S. tamariscina* may produce less apoplastic ROS and thus alleviates stress to cell membranes during water deficit. Choudhury et al. (2017) suggested that the ROS detoxification mechanisms within the cell walls are less effective than intracellular mechanisms, because they rely on low levels of ascorbate and glutathione, CuZn-superoxide dismutases, or cell wall peroxidases. This causes the accumulation of extracellular ROS which facilitates rapid systemic auto-propagating ROS waves (Choi et al., 2017; Choudhury et al., 2017). Despite being known as ROS-scavengers peroxidases also produce hydroxyl radicals from H_2_O_2_ which are capable of cleaving cell wall polysaccharides (**Figure 2**) (Fry, 1998; Passardi et al., 2004). Peroxidases in cell walls interact with polysaccharides and extensins, and supply phenoxy radicals for cell wall lignification and suberization (**Figure 2**) (Passardi et al., 2004; Tenhaken, 2015). Hence, peroxidases have dual functions: they contribute to wall loosening by releasing hydroxyl radicals and they boost wall stiffness by solidifying the extensin cross-linkages and supporting cell wall lignification and suberization (Novaković et al., 2018). Reinforcing the cell wall is an effective way to increase mechanical strength and to counteract increasing osmotic stress in response to dehydration. However, cell wall loosening is necessary for cell growth. In some resurrection plants the activities of peroxidases are highly increased upon rehydration, but do not change during dehydration (Sherwin and Farrant, 1998; Rodriguez et al., 2010; Deeba et al., 2016; Yobi et al., 2017). The limited activity of peroxidases may facilitate cell wall loosening and help reversible cell wall folding. In other resurrection plants, such as *X. viscosa*, peroxidases are up-regulated during dehydration but down-regulated upon rehydration, which is a prerequisite for cell wall stiffness under drought (Sherwin and Farrant, 1998; Ingle et al., 2007). Low-level substrates or decreased activity of peroxidases tend to generate hydroxyl radicals which lead to cell wall loosening and on the contrary high amounts of peroxidases, substrates, and ROS facilitate cell wall stiffness (Tenhaken, 2015).

### Receptor-Like Protein Kinases

The decoding of environmental cues and detection of cell wall perturbation under dehydration require special sensing mechanisms. Components of these sensors are members of RLK sub-families. RLKs generally consist of an extracellular domain, presiding over the perception of signals, a transmembrane region, and an intracellular kinase domain which triggers the downstream intracellular signaling (Ringli, 2010; Tenhaken, 2015; Novaković et al., 2018). Cell wall RLKs have been demonstrated to exert pivotal roles in plant development, growth and responses under various stresses, among which the well-characterized *Catharanthus roseus* protein kinase1-like receptor kinases (CrRLKs) and cell wall-associated protein kinases (WAKs) are candidates for cell wall integrity sensors (Novaković et al., 2018). In *A. thaliana*, there are 17 members of CrRLK (Lindner et al., 2012). Their involvement in Ca^2+^ signaling and ROS production during pollen tube growth, root hair elongation, or stress responses have been confirmed particularly for THESEUS1, FERONIA, and ANXUR (Hématy et al., 2007; Cheung and Wu, 2011; Denness et al., 2011; Boisson-Dernier et al., 2013; Feng et al., 2018). The FERONIA triggered-signaling is additionally regulated by a group of small peptides, RALFs (rapid alkalinization factors), which bind to FERONIA and also regulate a H^+^-ATPase and thus adjust the extracellular pH which subsequently determines activities of cell wall-remodeling enzymes (Murphy and De Smet, 2014). In analogy to FERONIA, the *C. plantagineum* WAK1 (CpWAK1) is a binding partner for the cell wall protein CpGRP1 (*C. plantagineum* glycine-rich protein1) (Giarola et al., 2016), and the *A. thaliana* WAK1 showed binding to both the AtGRP-3 protein and OGAs (**Figure 2**) (Decreux and Messiaen, 2005). WAKs are connected with turgor pressure as it was demonstrated that *Arabidopsis* plants silenced for *WAKs* had impaired cell expansion and reduced expression and activity of the vacuolar invertase (Kohorn et al., 2006). CpGRP1 and CpWAK1 accumulate in opposite directions upon dehydration and rehydration with more CpGRP1 and less CpWAK1 in desiccated samples compared to hydrated or rehydrated samples (Giarola et al., 2016). It was recently demonstrated that also CpGRP1 interacts with pectin and that the interaction is dependent on the homogalacturonan methylesterification status of pectin (Jung et al., 2019). The CpGRP1 protein binds stronger to homogalacturonan isolated from desiccated leaves than to homogalacturonan from hydrated leaves, where the degree of methylesterification is lower than in hydrated leaves. The data imply that both CpWAK1 and CpGRP1, or the CpWAK1-CpGRP1 complex participate in sensing changes in the cell wall organization and might trigger cell wall remodeling processes during dehydration (**Figure 2**).

### Hydroxyproline-Rich Proteins

Hydroxyproline-rich proteins are composed of highly O-glycosylated proteoglycans and exist in two forms in plants, one of which is insoluble and localized in the apoplast (Deepak et al., 2010; Shivaraj et al., 2018). The arabinogalactan proteins (AGPs) and extensins are two members of the hydroxyproline-rich protein family. Both strengthen the cell wall through crosslinking with other cell wall components and participate in signal transduction (Pilling and Höfte, 2003; Deepak et al., 2010; Ringli, 2010; Seifert and Blaukopf, 2010). AGPs have effects on cell expansion, growth, and pattern formation. The consensus structure of AGPs comprises a large carbohydrate moiety of type II arabinogalactans (β-(1,3)-galactan backbone decorated with arabinose and other polysaccharides in side chains) O-linked to the hydroxyproline (Hyp) residues of the polypeptide backbone (repetitive AlaHyp, SerHyp, and ThrHyp peptides) with an N-terminal signal sequence for secretion and a C-terminal glycosylphosphatidylinositol (GPI) lipid anchor tethering AGPs to the plasma membrane (**Figure 2**) (Knoch et al., 2014; Lamport et al., 2014; Tan et al., 2018). AGPs form a diverse class of proteins due to variable compositions of the peptide backbone and the carbohydrate moieties. Moore et al. (2006; 2013) supported the notion that AGPs serve as “plasticizers” to maintain cell wall flexibility during desiccation in the resurrection species *Mohria caffrorum*, *M. ﬂabellifolia*, *C. plantagineum*, the grass-like *Xerophyta* spp., and the grass *Erograstis nindensis*. OGAs released from AGPs may facilitate to maintain intracellular osmotic pressure during dehydration according to the analysis of AGP genes in rice (Ma and Zhao, 2010). However, the function of AGPs is probably not only restricted to the release of OGAs as signaling molecules, but AGPs may act as sensors (Ringli, 2010; Seifert and Blaukopf, 2010; Lamport et al., 2014; Novaković et al., 2018).

Extensins are characterized by the repetitive SerHyp4 and SerHyp2 motif and the Tyr-Lys-Tyr sequence with Ser residues decorated with single galactose and minor arabinogalactan moieties attached to hydroxyproline residues (Shivaraj et al., 2018; Tan et al., 2018). The self-assembling extensins usually function as positively charged scaffolds, interacting with de-methylesterified pectin *via* adsorption (Cannon et al., 2008; Valentin et al., 2010). Extensins facilitate the crosslinking of rhamnogalacturonan-II *via* borate and also contribute to ion-regulated cell wall integrity (Chormova and Fry, 2016; Tan et al., 2018). Besides a structural role, the proline-rich extensin-like receptor kinases have an effect on cell wall signal transduction. The T-DNA mutant *perk4* (defective extensin-like receptor kinase) displayed decreased sensitivity to ABA and lower [Ca^2+^]_cyt_ and Ca^2+^ channel currents upon ABA treatment (Bai et al., 2009). This supports a role of an extensin-like receptor kinase in ABA signaling and Ca^2+^ homeostasis.

### Cell Wall Modifying Proteins

The remodeling of the polysaccharide composition under dehydration or rehydration is catalyzed by different cell wall modifying proteins and enzymes. Understanding the activity and regulation of these proteins and enzymes is crucial to decipher the folding process. Cell wall modifying enzymes are a group of cell wall proteins regulating cell wall composition and rheology. Here, three members of cell wall modifying proteins and their functions in resurrection plants are described.

### Expansins

Expansins are hypothesized to prompt acid-induced growth and cell wall remodeling under abiotic stress by disrupting hydrogen-bonds between xyloglucan and cellulose microfibrils without lytic activity (**Figure 2**) (Cosgrove, 2015; Tenhaken, 2015). Plant expansins fall into two major families: α-expansins and β-expansins according to phylogenetic analyses (Cosgrove, 2015). The expression and activity of α-expansins were studied in *C. plantagineum* leaves (Jones and McQueen-Mason, 2004). Among the three expansin transcripts the *CplExp1* transcript level was correlated with expansin activity which increased in the early stages of dehydration and rehydration corresponding to cell wall extensibility (Jones and McQueen-Mason, 2004). This suggests a role of expansin-induced wall extension in the early stages of dehydration and rehydration. However, dehydration tends to alkalize the apoplast which leads to the question how the activity of acid-activated expansins can be triggered upon dehydration (Geilfus, 2017).

### Xyloglucan Endotransglucosylases/Hydrolases

Apart from expansins, the xyloglucan endotransglucosylases/hydrolases (XTHs) are other candidates for unzipping the hemicellulose (xyloglucan)-cellulose network *via* hydrolysis or transglucosylation to increase the cell wall extensibility (**Figure 2**) (Sasidharan et al., 2011; Tenhaken, 2015). In contrast to expansins, XTHs exhibit two activities: irreversible xyloglucan hydrolysis (XEH) and reversible xyloglucan endotransglucosylation (XET), suggesting roles for XTHs in cell wall loosening and re-assembling (Rose et al., 2002). Transgenic *A. thaliana* and tomato plants overexpressing a xyloglucan endotransglucosylase/hydrolase CaXTH3 from hot pepper showed enhanced tolerance to salt and dehydration stress (Cho et al., 2006; Choi et al., 2011). In the resurrection plant, *Haberlea rhodopensis*, one putative XTH gene HrhDR35 was up-regulated during early dehydration to desiccation and rehydration (Georgieva et al., 2012), which corresponds to expansin expression in *C. plantagineum* (Jones and McQueen-Mason, 2004). Based on these observations it is suggested that XTHs contribute to improve dehydration tolerance through increasing wall extensibility and cell wall reconstruction after stress relief in both desiccation tolerant and desiccation sensitive plants. However, XTHs may not always contribute to cell wall extensibility as was shown for cell walls of ripening tomato fruit (Saladié et al., 2006)

### Pectinmethylesterases

The degree of pectin methylesteriﬁcation is an important factor for cell wall structure and has an effect on cellular growth and cell wall responses during dehydration (Wolf et al., 2009). Pectinmethylesterases de-methylesterify pectin and thus generate negatively charged pectin (**Figure 2**). This reaction is affected by the apoplastic pH and the degree of methylesteriﬁcation of galacturonic acid (Micheli, 2001; Wolf et al., 2012). The released pectin transfers Ca^2+^ to promote the formation of egg-box pectin gelatin which enhances the mechanical stability (Wolf et al., 2012; Voxeur and Höfte, 2016). Under dehydration the cell wall texture is presumably modified by the egg-box gelatin through activating pectinmethylesterases and inhibiting expansins due to the increased pH of the apoplast (**Figure 2**) (Wolf et al., 2012). Upon dehydration de-methylesterified pectin increases in the cell wall of *C. wilmsii*, *C. plantagineum,* and *L. brevidens* which is probably due to pectinmethylesterase activities during dehydration (Vicré et al., 1999; Vicré et al., 2004a; Jung et al., 2019).

The above-described cell wall proteins are not sufficient to explain the cell wall behavior during dehydration/rehydration in resurrection plants. Analyses of genome sequences have identified several other cell wall proteins in resurrection plants (Giarola et al., 2015). Giarola et al. (2015; 2016) identified a cysteine-rich protein localized in the apoplast and down-regulated during dehydration but up-regulated during rehydration, while the expression level of the CpGRP1 protein was enhanced during desiccation, which is consistent with the dehydration-induced GRP1 from *Boea hygrometrica* (Wang et al., 2009). Also aquaporins and plasma membrane intrinsic proteins accumulated upon dehydration or in the presence of ABA in *C. plantagineum*, suggesting that water channels are associated with ABA signaling during dehydration (Mariaux et al., 1998).

## Changes in Cell Wall Transcriptomes Upon Dehydration in Resurrection Plants

In the past 10 years transcriptome-wide changes upon dehydration and rehydration have been reported for the dicot resurrection species *C. plantagineum* (Rodriguez et al., 2010), *H. rhodopensis* (Gechev et al., 2013), *M. flabellifolia* (Ma et al., 2015), and *Boea hygrometrica* (Xiao et al., 2015; Zhu et al., 2015), and for the monocot resurrection species *Oropetium thomaeum* (VanBuren et al., 2015) and *Sporobolus stapfianus* (Yobi et al., 2017). This information can be used to identify genes which are related to cell wall compartments. This will allow a comprehensive study of the molecular mechanisms which are activated to adapt the cell walls to the reducing cell volume caused by water loss in resurrection plants.

The analysis of transcriptome data showed that several cell wall-related genes which are involved in different processes such as the regulation of cell wall plasticity and cell wall dynamics, catabolic processes, and cell wall organization are differentially modulated upon dehydration thus suggesting the importance of cell wall remodeling during the acquisition of desiccation tolerance (Rodriguez et al., 2010; Gechev et al., 2013; Xiao et al., 2015; Zhu et al., 2015). One main obstacle to the interpretation of RNA expression data resides in the fact that genes encoding cell wall modifying enzymes belong to large gene families and often different enzyme isoforms in these families are differently regulated upon dehydration. **Table 1** summarizes dehydration-induced changes in the expression of genes encoding cell wall modifying proteins and enzymes which were reported for resurrection species. A good example of enzyme isoforms which display an opposite expression upon dehydration is represented by xyloglucan endotransglucosylases (XTHs) in *H. rhodopensis*. Transcriptome data showed that several XTHs isoforms are down-regulated during dehydration (Gechev et al., 2013) (**Table 1**) but the presence of a dehydration-induced XTH was previously identified by cDNA-AFLP experiments (Georgieva et al., 2012). Genes encoding XTHs, expansins, pectinmethylesterases, and pectinacetylesterases are abundant in hydrated leaves of *C. plantagineum* and down-regulated upon dehydration (Rodriguez et al., 2010). The stage where the maximum transcript expression is registered can also be hardly used as indicator for protein activity as this can be affected by additional factors, e.g., the binding with specific inhibitors, changes in the apoplastic pH, the substrate accessibility, and/or the accumulation of ROS upon dehydration.

**Table 1:** Dehydration-induced expression changes of cell wall enzymes which were reported for resurrection species.

Species	Cell wall enzymes	Expression	Reference
*Craterostigma plantagineum*	Xyloglucan endotransglucosylases, pectin methylesterases and pectin acetylesterases	Downregulated upon dehydration	(Rodriguez et al., 2010)
*Sporobolus stapfianus*	Endo-beta-mannanase, beta- mannan endohydrolase, beta-D-glucan exohydrolase, glucan endo-1,3-beta-glucosidase, feruloyl esterase, glycosyl-transferases	very abundant in late dehydration/desiccation (RWC ≤ 30%)	(Yobi et al., 2017)
*Sporobolus stapfianus*	Cell wall-associated hydrolases	abundant in early stage of dehydration (80% RWC)	(Yobi et al., 2017)
Sporobolus stapfianus	Cellulose synthases, lichenase, glucan endo-1,3-beta-glucosidase, anthocyanidin 5,3-O-glucosyltransferase	Downregulated upon dehydration	(Yobi et al., 2017)
*Haberlea rhodopensis*	Xyloglucan endotransglucosylases, pectin esterases and pectate lyases	Downregulated upon dehydration	(Gechev et al., 2013)
*Haberlea rhodopensis*	Laccase	Accumulated in late dehydration/desiccation	(Gechev et al., 2013)

Our knowledge of structural changes in cell walls of dehydration sensitive species upon dehydration stress is limited and thus it is difficult to identify which mechanisms are specific for cell wall folding in resurrection species. Increased cell wall extensibility is observed upon dehydration in the resurrection species *Craterostigma* and it appears to be essential for cell wall folding and survival (Jones and McQueen-Mason, 2004). Conversely, sensitive plants subjected to drought stress tend to increase the stiffness of their cell walls (Lu and Neumann, 1998; Tenhaken, 2015). Expansins and XTHs have been proposed to be good candidates to increase cell wall extensibility in resurrection species. Additionally, other cell wall modifying proteins or enzymes, e.g., pectinmethylesterases are emerging as possible modulators of cell wall stiffness by acting on the methylesterification level of pectin. Finally, transcriptome data suggest the involvement of several classes of cell wall modifying enzymes and cell wall modifying enzyme inhibitors but the sole transcript data are far from providing a clear picture of how the different classes of cell wall proteins from these species are recruited and coordinated to achieve cell folding in resurrection species.

## Conclusions

Cell wall remodeling is a pivotal drought tolerance mechanism for plants (Tenhaken, 2015), which includes two opposite effects: stiffening and loosening. Both effects contribute to the ability to overcome mechanical stress, while stiffening preferentially occurs in desiccation sensitive plants and loosening is essential for cell wall folding in resurrection plants. Maintaining the integrity of cell walls during dehydration and rehydration in resurrection plants involves many components ranging from changes in polysaccharide composition to differential RNA expression. The activation of pathways leading to more flexible components on the one hand and adding more stability to the cell wall on the other hand, suggests a tightly controlled folding process during dehydration which finally keeps the plasmalemma and the photosynthetic apparatus intact in resurrection plants.

## Author Contributions

PC, NJ, and VG wrote the manuscript. DB and VG supervised the work and corrected the manuscript.

## Funding

PC acknowledges a fellowship from CSC and part of the work was supported by the DFG project Smartwall (BA 712-18-1).

## Conflict of Interest

The authors declare that the research was conducted in the absence of any commercial or financial relationships that could be construed as a potential conflict of interest.

## Abbreviations

OGAs, Oligosaccharides; WAKs, Wall-associated protein kinases; ROS, Reactive oxygen species; RLKs, Receptor-like kinases; ABA, abscisic acid; GRP, Glycine-rich protein; AGPs, Arabinogalactan proteins; XTHs, Xyloglucan endotransglucosylases/hydrolases.
